# Description of a Case of Frontal Pain Occurring in a Patient in the Marine Hospital, Charleston, S. C., Relieved by Large Doses of Hydriodate of Potassa

**Published:** 1856-04

**Authors:** F. Peyre Porcher


*Description of a Case of Frontal Pain occurring in a patient in the Marine Hospital, Charleston, S. C., relieved by large doses of Hydriodate of Potassa.* By F. Peyre Porcher, M. D.

During an attendance of some eight months as Physician to the Marine Hospital, *vice* Dr. Cain absent from sickness, I have been enabled to note several results consequent upon the use of Hydriodate of Potassa in very large doses.

I am indebted to Mr. Carswell, a student of Medicine in the Medical College of South Carolina, for furnishing me with an abstract from the daily notes of the case taken at the time, from the Hospital Book. It is an interesting one, as it shows the relief afforded by the use of this medicine afier various powerful opiates had completely failed. It is instructive, too, in connection with the Materia Medica—quite a number of articles being made use of during the treatment. There are, also, one or two points connected with the diagnosis worthy of attention; the failure of certain agents reflecting light upon the latter by *a posteriori* reasoning.

What the medical man at the present day covets most, is a knowledge of the precise influence and effects, whether favorable or not, of certain agents, upon the human system.

In this case, for example, he is instructed, by being informed that five grains of solid opium, on one occasion, and two grains of morphia, on another, taken duriug the course of a few hours, gave no relief to pains which were before believed to be neuralgic in character. That no local anodynes, even of the most potent kinds, were productive of any benefit, when rubbed into the skin; that all these failing, another substance produced its specific effects, and in curing the patient, taught the attendant what was probably the true nature of the affection. It is only by careful scrutiny of recorded observations as to the effects of drugs, that any valuable contribution to our knowledge of the Materia Medica will be obtained. The process is a slow one, depending upon the gradual accretion of facts—uniformity in the results, by repetition of the use of the same means, cannot always be depended upon—yet, the progress, after a series of years, is encouraging.

Mulligan, a sailor, aged 40, complained of pain in the head, following a severe cold contracted at sea some five years since. Though an inmate of several Hospitals, at different times, this persisted; now and then abating somewhat in violence, and generally, if not always, being more severe at night. He stated, that at one period a salivation had freed him from suffering for six weeks.

Mulligan entered the Marine Hospital the 27th July, 1855, and was discharged September 27th, completely relieved; which I hope has been permanent. He complained of intense and severe pains in the frontal bones and in the arm, coming on in the evening, and persisting during the night. There was also pain about the abdomen, which very soon disappeared upon the use of a blister. The pains complained of were always excessive, and seemed to require active interference. It will be seen how ineffectual the opiates, and indeed the whole class of neurotics proved to be. The patient had had gonorrhœa, but never chancre; nor were there any cicatrices either upon or around the organs of generation, nor in the urethra, which was carefully inspected. He had never had intermittent fever. As several of his symptoms, however, may have had their origin in rheumatism, the following combinations were at first employed, but without avail.

Before commencing their recital, I must offer some apology for such an apparently experimental course; but the prescriptions were all made with a view to meet certain prominent indications. The negative testimony here is almost, if not quite as interesting as the positive, as we will observe what large doses were used, often too, of powerful and active substances.

*July 28th.*—℞. Sal. Rochelle, ℥ss; tinc. hyoscyam., ʒi; aqua, ℥iv; extr. hyoscyam., grs.v. M. Take at bed time.

*July 29th.*—No improvement. ℞. Sulph. of quinine, grs.v; tinct. aconite seeds, gtt.x. M. To be taken three times a day, with the use of mustard foot baths and warm herb teas.

*August 1st.*—Blister on the loins. Above prescription continued to

*August 4th.*—Blister over the region of the chest.

*August 6th.*—℞. Tinct. cinchona, ʒi; sulph. quinine, grs.v; tinct. aconite, gtt.x. M. To take four times a day.

Tint. cinchona, with the tinct. of sanguinaria was also employed. The preparations of guaiac. and colchicum, in the following formula, were used rather empirically, but with some view to meet any rheumatic element in the disease. It was ineffectual. ℞. Tinct. guaiac., tinct. colchicum, aa ℥ss: sulph. morphia, grs.ii; bi-carb. potash., ʒss; sulph. quinine, grs.x; water, ℥iv. A tablespoonful three times a day.

Astley Cooper having recommended the mezereon root (*Daphne mezereon*) as a remedy in nocturnal pains, I determined to try it in this obstinate case, and accordingly strong decoctions were employed for several days, but ineffectually.

The following formula, containing strychnine and other neurotics was then resorted to with similar unsuccess: ℞. Strychnia, gr.1-16; bellad. extr., gr.$\frac{1}{4}$; hyoscayamus, gr.$\frac{1}{2}$. Three pills, each containing the above, employed three times a day. Continued from August 17th to 21st.

On the 21st of August the patient took ten grains of quinine before the pains came on.

*August 22d.*—He was ordered: ℞. Sulph. morph., gr.$\frac{1}{2}$; repeating it every half hour, in order to see what effect large doses of the opiate would have. Two grains of morphia were given during the course of the night without relief.

On the next day the patient was prescribed cod liver oil and iron: ℞. Cod liver oil, ʒi; mur. tinct. iron, gtt.vii, three times a day; and on the 24th ten drops of arom. sulph. acid were added to each dose. I then determined to try narcotic ointments, and the following was ordered to be rubbed on the forehead repeatedly: ℞. Morphia, grs x; pulv. opium, grs.xx; laudanum, ʒii; extract bellad., ʒii: extract stramonium, ʒii. These proving, likewise, ineffectual, the tincture of aconite (seeds) was rubbed into the skin, upon the brow and forehead.

On the next evening Mulligan again took two grains of morphia, in half grain doses, every hour. The pain was lessened, but there was no sleep.

On the 1st September a blister was applied to the nape of the neck, and on the 22d he took a tablespoonful, three times a day, of syrup of sarsaparilla, with two grains of corrosive sublimate to the bottle. This was continued for three days, and on the evening of the day subsequent five grains of powdered opium were swallowed in a few hours. It gave more rest than the morphine.

Hydriodate of potassa, in ten grain doses, three times a day, not being attended with any benefit, on September 11th he commenced with grs.xv, three times a day.

*September 13th.*—It was increased to grs.xxx, three times a day.

*September 18th.*—Discontinued potash for two days. It was then resumed, having been found to give complete relief, and used until the 26th, when the patient complained of some pain and tightness about the abdomen, with a salty taste in the mouth, and a running at the nose, which I considered as indications for its discontinuance in such doses.

Since the 13th he has enjoyed a complete cessation of pain, and he left the Hospital entirely relieved.

I should add that this man has been passing portions of tape-worm continually, he says, for many years. When he left, they were occasionally voided, but in no respect did they seem to be connected with the pains of which he suffered.

Now I explain, in this instance, the curative influence of the Hydriodate of Potassa (in maximum doses, it will be remembered, for the drug administered in small quantities was ineffectual,) by the theory suggested by Williams, who first discovered and enforced its great value in treating tertiary syphilis. This compels us to regard Mulligan's case as one of exostosis, or periostitis, whether syphilitic or not is doubtful. Williams discovered that it was specific in the cure of nocturnal and neuralgic syphilitic pains, by inducing absorption of the matter deposited between the periosteum and the bone, the pressure produced by, or the mere presence of which, gives rise to the pain.

Hydrodate of Potassa is, indeed, a true specific, and its application in this way is very properly acknowledged to be one of the most important discoveries of the last half century. The drug is specific in removing, by encouraging the action of the absorbants, the deposit which gives rise to the pain, and which, allowed to increase, constitutes nodes. Dr. Budd, and recent writers (see late Medical Journals) counsel us to employ it before the third stage of syphilis has shown itself, in order to ward it off, by preventing the deposit of lymph. Williams, it seems, made this discovery after a series of experiments with a number of different articles, with the expressed intention to find some one capable of accomplishing the object in view. Every day experience shows how completely successful he was.

Strange to say, some writers discuss gravely the toxicological effects of three grain doses.

In reading over a number of notes, taken during the year 1853, at M. Nélaton's clinique, in Paris, I find that he calls special attention to some of the variable and unforseen effects of this drug; among these, he cites salivation, a blue line in the conjunctiva, etc.

During my absence from the Hospital for a week at Christmas, a friend prescribed to three of the patients there 60 grains a day. In one, O'Harra, it was followed by diarrhœa and emaciation, which still obstiinately continues. In another, Johnston, I repeated the prescription of sixty grains a day, in twenty grain doses, for ten days, without the slightest injurious or appreciable effects, except to relieve the nocturnal pains in tertiary syphilis, of which he is suffering. Upon discontinuing the medicine, in order to test the relative value of tincture of iodine applied externally, we found that they returned after three days. We were obliged to have recourse again to the Hydriodate of Potassa, gradually increasing it from 10 grains, three times a day. He now (February 7th, 1856,) finds relief again from the pains. In medium doses it is a drug repeatedly prescribed at the Hospital. The present head nurse at the Hospital informs me that he himself took 40 grains a day for twenty days, without untoward symptoms, except a feeling of fullness about the head, and symptoms of catarrh.—*Chas. Med. and Surg. Journal.*

